# Prognostic value of memory B cell subpopulation in patients with chronic lymphocytic leukemia

**DOI:** 10.1007/s10238-025-02002-5

**Published:** 2025-12-24

**Authors:** Aviwe Ntsethe, Bongani Brian Nkambule

**Affiliations:** 1 Department of Human Physiology, Nelson Mandela University, Private Bag X77000, Gqeberha, 6031 South Africa; 2 School of Laboratory Medicine and Medical Sciences (SLMMS), College of Health Sciences, University of KwaZulu-Natal, Durban, South Africa

**Keywords:** Chronic lymphocytic leukaemia, Memory b cells, International prognostic index for CLL, Beta-2 microglobulin, Rai stage, CLL-IPI

## Abstract

Chronic lymphocytic leukemia (CLL) is characterized by aberrant B-cell receptor (BCR) signalling and resistance to apoptosis. Patients with CLL present varied responses to BCR-targeted therapies, this emphasises the need for personalised treatment strategies. The immunophenotype of B cell in patients with CLL may offer valuable insights into disease heterogeneity and immunotherapeutic strategies. In this study, we investigated the prognostic value of B cell subsets in relation to clinical parameters such as Rai stage, β₂-microglobulin (B2M) levels and the Chronic Lymphocytic Leukemia International Prognostic Index (CLL-IPI) in untreated patients with CLL. The levels B cell subsets were measured using multicolor flow cytometry. Interestingly, patients with CLL had significantly increased levels of naïve B cells (*p* < 0.0001). As expected, patients with CLL had significantly decreased levels of marginal zone B cells (*p* = 0.0059), non-class switched memory B cells (*p* = 0.0010), and class switched memory B cells (*p* < 0.0001). Moreover, after adjusting for age and sex, class-switched memory B cells were positively associated with the Rai stage (β = 0.1455, *p* = 0.0466). In contrast, non-class switched memory B cells inversely correlated with the Chronic Lymphocytic Leukemia International Prognostic Index (*r* = -0.79, *p* = 0.004). These findings suggest that memory B-cell subset profiles may provide valuable prognostic significance in patients with CLL.

**Supplementary Information:**

The online version contains supplementary material available at 10.1007/s10238-025-02002-5.

## Introduction

Chronic lymphocytic leukemia is a lymphoproliferative disorder driven by aberrant B-cell receptor (BCR) signalling, primarily due to overexpression of B-cell lymphoma 2 (Bcl-2). This leads to the accumulation of mature functionally incompetent B-cells in peripheral blood, bone marrow, lymph nodes, and the spleen [1, 2]. The development of Bcl-2 inhibitors such as venetoclax, which induce apoptosis of malignant cells has led to improved patient outcomes [3]. Furthermore, standard treatment strategies for patients with CLL often include anti-CD20 monoclonal antibodies such as rituximab, obinutuzumab and ofatumumab [4–6]. However, CLL remains a heterogeneous disease characterized by a diverse clinical course [7]. Patients with CLL can live for years without the need for intervention (CLL-WONT) [8].

Patients with CLL are classified according to Rai staging, as low risk (stage 0), intermediate risk (stages I and II), or high risk (stages III and IV) [9]. The CD38 is one of the most frequently reported prognostic markers in patients with CLL. The expression of ≥ 30% CD38 positive malignant cells is associated with poor overall survival and resistance to immunotherapy [10]. In addition, β2-microglobulin (B2M) is one of the most robust and well-established independent prognostic markers in CLL due to its strong association with disease progression and overall survival [11]. However, the incorporation of various prognostic markers in patient risk stratification remains a challenge.

The B cell function is dysregulated in patients with CLL, and this contributes to immune dysfunction and disease progression [12, 13]. Although CLL is fundamentally a malignancy of B cells, surprisingly few studies have investigated the prognostic value of B cell subsets in patients with CLL. B cell subsets such as naïve, and regulatory B cells are known to be altered in patients with CLL, with evidence suggesting immune dysfunction, impaired antibody responses, and increased susceptibility to infections [14]. Moreover, there is a differential expression of immune checkpoint molecules such as programmed cell death protein 1 (PD-1), programmed death ligand 2 (PD-L2), and cytotoxic T-lymphocyte associated protein 4 (CTLA-4) across B cell subsets, indicating a major role played by these cells in disease progression [15]. The aim of this study was to investigate the prognostic value of B cell subsets profiles in patients with CLL.

## Methods and materials

### Patient recruitment

Participants were recruited at the King Edward VIII Hospital, Durban, South Africa from July 2019 to May 2022 as previously described [16]. The study was approved by the University of KwaZulu-Natal Biomedical Research Ethics Committee, South Africa (Ethical approval number: BE456/18). All participants provided written informed consent.

### Inclusion and exclusion criteria

The study included treatment naïve patients with CLL and healthy controls without any clinical signs of infection. Patients undergoing treatment for CLL were excluded.

### Sample collection

Five milliliters (5 mL) of venous blood was collected from consenting participants via venipuncture into 6 mL ethylenediaminetetraacetic acid (EDTA) tubes (BD Bioscience, San Jose, CA, USA). The samples were then transported at room temperature (20–25 °C) from the hospital to the laboratory.

### Isolation of peripheral blood mononuclear cells (PBMCs)

Peripheral blood mononuclear cells (PBMCs) were isolated from whole blood using the density-gradient centrifugation with Ficoll-Paque PLUS (Amersham, Biosciences, Uppsala, Sweden), as previously described [16, 17]. Briefly, 4 mL of whole blood was layered on a Ficoll-Paque PLUS gradient (Sigma-Aldrich, Roedermark, Germany). Samples were then centrifuged at 400 × g for 40 min at 20 °C. Isolated PBMCs were collected and stored at − 80 °C.

### T cell depletion and B cell isolation from peripheral blood mononuclear cells

To enrich the B cell population from the isolated PBMCs, T cell depletion followed by positive B cell selection was performed using the BD IMag™ isolation system (BD Biosciences, San Jose, CA, USA) as we previously described [16]. Briefly, 50 µL of PBMCs were incubated with 5 µL of a biotinylated human T lymphocyte enrichment cocktail (BD Biosciences, San Jose, CA, USA) for 15 min at room temperature. After incubation, 50 µL of streptavidin-coated magnetic particles were added to the T cell-depleted PBMCs and incubated for an additional 30 min at room temperature. The samples were then resuspended in 1 mL of 3.2% sodium citrate buffer and placed on the BD IMag™ magnet for 8 min. The isolated B cells were subsequently resuspended in 100 µL of phosphate-buffered saline (PBS) and stored at − 80 °C.

### Measurements of B cell subsets

**Fig. 1:**
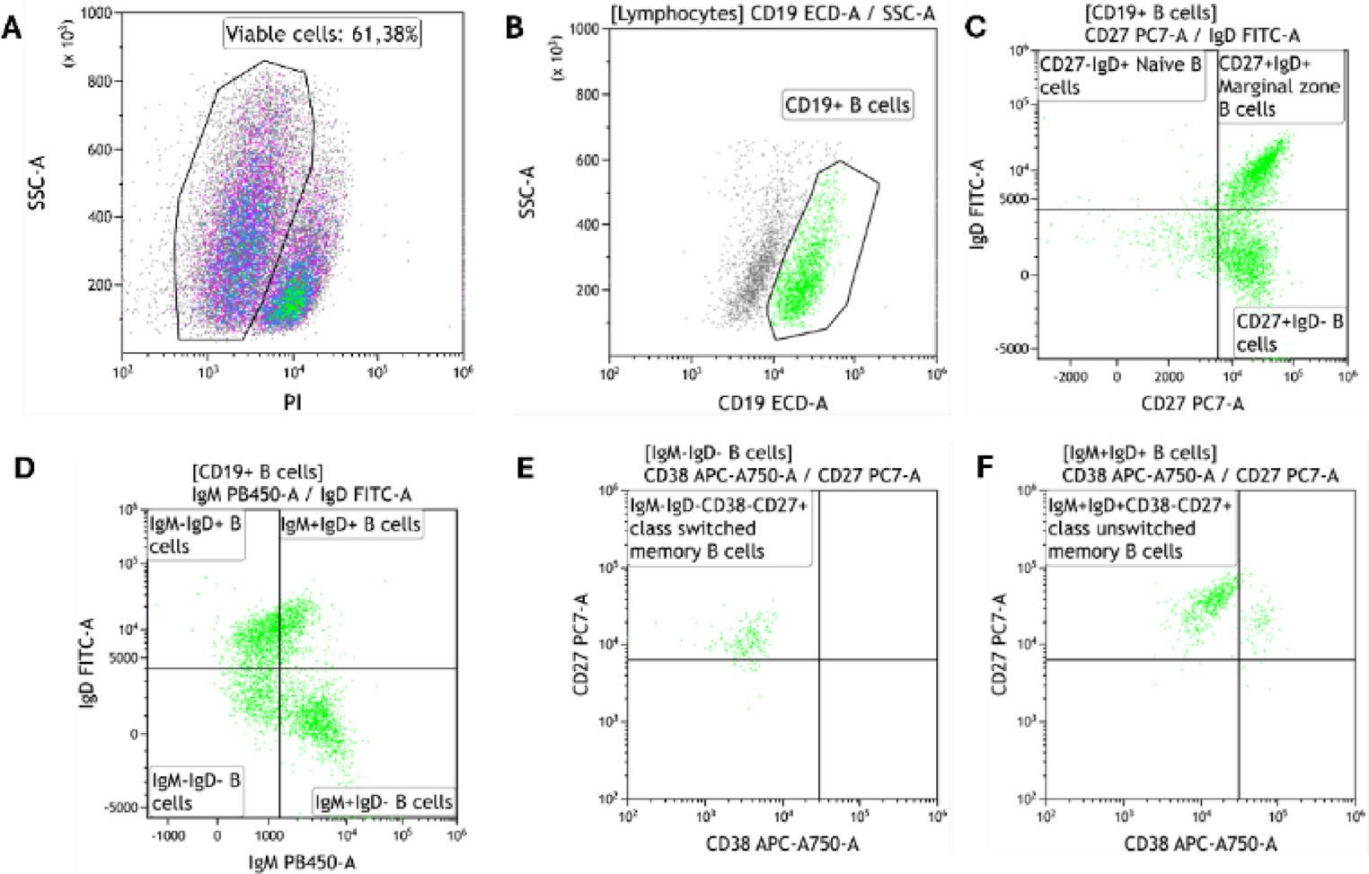
Gating strategy. (**A**) illustrates the gating of viable cells using propidium iodide (PI) negative events. (B) illustrates the gating of B cells based on side scatter (SSC) and CD19 expression. (**C**) illustrates the gating of naïve B cells defined as CD19^+^CD27^−^IgD^+^ events, marginal zone B cells defined as CD19^+^CD27^+^IgD^+^ events and CD19^+^CD27^+^IgD^−^ memory B cells, respectively. Figure **D** illustrates the gating of CD19^+^IgM^−^IgD^+^, CD19^+^IgM^+^IgD^+^, CD19^+^IgM^−^IgD^−^ and CD19^+^IgM^+^IgD^−^ B cells, respectively. (**E**) illustrates the gating of class switched memory B cells defined as CD19^+^CD27^+^CD38^−^IgM^−^IgD^−^ events. (**F**) illustrates the gating of class unswitched memory B cells defined as CD19^+^CD27^+^CD38^−^IgM^+^IgD^+^ events

The B cell subsets were determined using the DuraClone IM B cell panel (Beckman Coulter, Brea, California, USA) on a six colour DxFLEX flow cytometer and analysed using KALUZA software (Beckman Coulter, Brea, California, USA). The DURAClone panel comprised of CD45- Krome Orange, CD19-ECD, CD27-PC7, CD38- APC750, IgM- Pacific Blue, IgD-FITC. While CD24 and CD21 are included in the DURAClone IM B Cell panel and are valuable markers for distinguishing transitional and atypical B-cell populations, these were not included in the current study, as our analysis was designed to focus on major B-cell subsets, specifically naïve and memory B cells. Future studies incorporating these markers may provide a more detailed characterization of transitional and atypical B-cell compartments in CLL. Positive staining for propidium iodide (PI) (Sigma-Aldrich, Roedermark, Germany) was used to distinguish between viable and non-viable cells (Fig. 1A). Lymphocytes were defined as CD45^+^/low SSC events. We acquired at least thousand CD19^+^ events (B cells) (Fig. 1B). Naïve B cells were defined as CD19^+^CD27^−^IgD^+^ events, marginal zone B cells defined as CD19^+^CD27^+^IgD^+^ events and memory B cells as CD19^+^CD27^+^IgD^−^, respectively (Fig. 1C). We further determined CD19^+^IgM^−^IgD^+^, CD19^+^IgM^+^IgD^+^, CD19^+^IgM^−^IgD^−^ and CD19^+^IgM^+^IgD^−^ B cells, respectively (Fig. 1D). Class switched memory B cells were defined as CD19^+^CD27^+^CD38^−^IgM^−^IgD^−^ events (Fig. 1E) and non-class switched memory B cells were defined as CD19^+^CD27^+^CD38^−^IgM^+^IgD^+^ events (Fig. 1F). A detailed gating hierarchy used to identify B-cell subsets from PBMCs is provided in the supplementary material (Figure S1).

### Determining the concentration of plasma soluble Beta-2 microglobulin (B2M)

Plasma levels of beta-2 microglobulin (B2M) were quantified using a human enzyme-linked immunosorbent assay (ELISA) (ThermoFisher Scientific, Waltham, MA, USA), following the manufacturer’s protocol.

### Sample size Estimation

We calculated the minimum sample size required to detect a large effect size (d = 1.52) in B cell subset levels between patients with CLL and healthy controls. Assuming 85% statistical power and a significance level (α) of 0.05, a two-tailed unpaired t-test indicated that a minimum of ten (*n* = 10) patients with CLL and six (*n* = 6) healthy controls were needed. All sample size estimations were performed using G Power version 3.1.94 (Universität Düsseldorf, Germany).

### Statistical analysis

Data distribution was assessed for normality, and statistical analysis choices were made accordingly. An unpaired Student’s t-test was performed to compare parametric data between the two groups and reported as the mean and standard deviation (SD). Mann-Whitney U test was performed to compare non-parametric data between the two groups and reported as the median and interquartile range (IQR). To investigate the association between B cell subsets and plasma β2-microglobulin (B2M) levels, we performed multiple linear regression analysis using Python (v3.10) with the statsmodels, scipy, and seaborn libraries. The model was adjusted for age and sex to account for potential confounding. Correlation between each B cell subset and B2M was also assessed using Pearson correlation coefficients (r), and linear regression plots were generated with best-fit lines and 95% confidence intervals. Regression coefficients (β), standard errors (SE), and p-values were reported. A p-value < 0.05 was considered statistically significant. All statistical analysis was performed using GraphPad Prism version 8 software, (GraphPad Software Inc., San Diego, CA, USA) and Python (v3.10).

## Results

### Patients characteristics

This study is comprised of 11 patients with CLL, with a mean age of 63.45 ± 11.97 years. The study included 45.45% females and 54.55% males (Table 1).

**Table 1:** Participants’ characteristics and hematological parameters

	Control (*n* = 11)	Patients with CLL (*n* = 11)	*p*-Value
Age (Years)	55.73 ± 16.14	63.45 ± 11.97	0.2167
Male, n (%)	54.55	54.55	
Female, n (%)	45.45	45.45	
White blood cell count (10^3^ µL)	5.2 ± 1.43	162.5 ± 160.9	0.0043
Red blood cell (10^6^ µL)	4.72 ± 0.99	2.17 ± 1.13	0.0004
Haemoglobin (g/dL)	14.04 ± 3.98	8.23 ± 1.63	0.0012
Platelets (10^3^ µL)	200.9 ± 68.49	199.0 ± 193.2	0.9759

### Diagnostic profiles

Patients with CLL were diagnosed based on the standards from the International Workshop on Chronic Lymphocytic Leukaemia [18]. The clinical CLL stage was determined according to the Rai classification system [9], 45.45% were on stage IV, 36.36% were on stage III and 18.18% were on stage II (Table 2). The cohort consisted of most patients with 13q14 deletion (54.55%), followed by 11q22 deletion (27.27%) and 17p13 deletion (18.18%) (Table 2). The biological characteristics of each patient with CLL are summarized in the supplementary material (Table S1).

**Table 2:** Diagnostic profile of patients with CLL (*n* = 11)

Clinical Parameters	
**RAI Staging**	
II, n (%)	2 (18.18)
III, n (%)	4 (36.36)
IV, n (%)	5 (45.45)
**Deletions**	
11q22, n (%)	3(27.27)
13q14, n (%)	6 (54.55)
17p13, n (%)	2 (18.18)
**CLL-IPI**	
Low risk, n (%)	7 (63.64)
Intermediate risk, n (%)	2 (18.18)
High risk, n (%)	2 (18.18)

### Increased levels of naïve B cells and decreased levels of memory B cells in patients with CLL

**Fig. 2:**
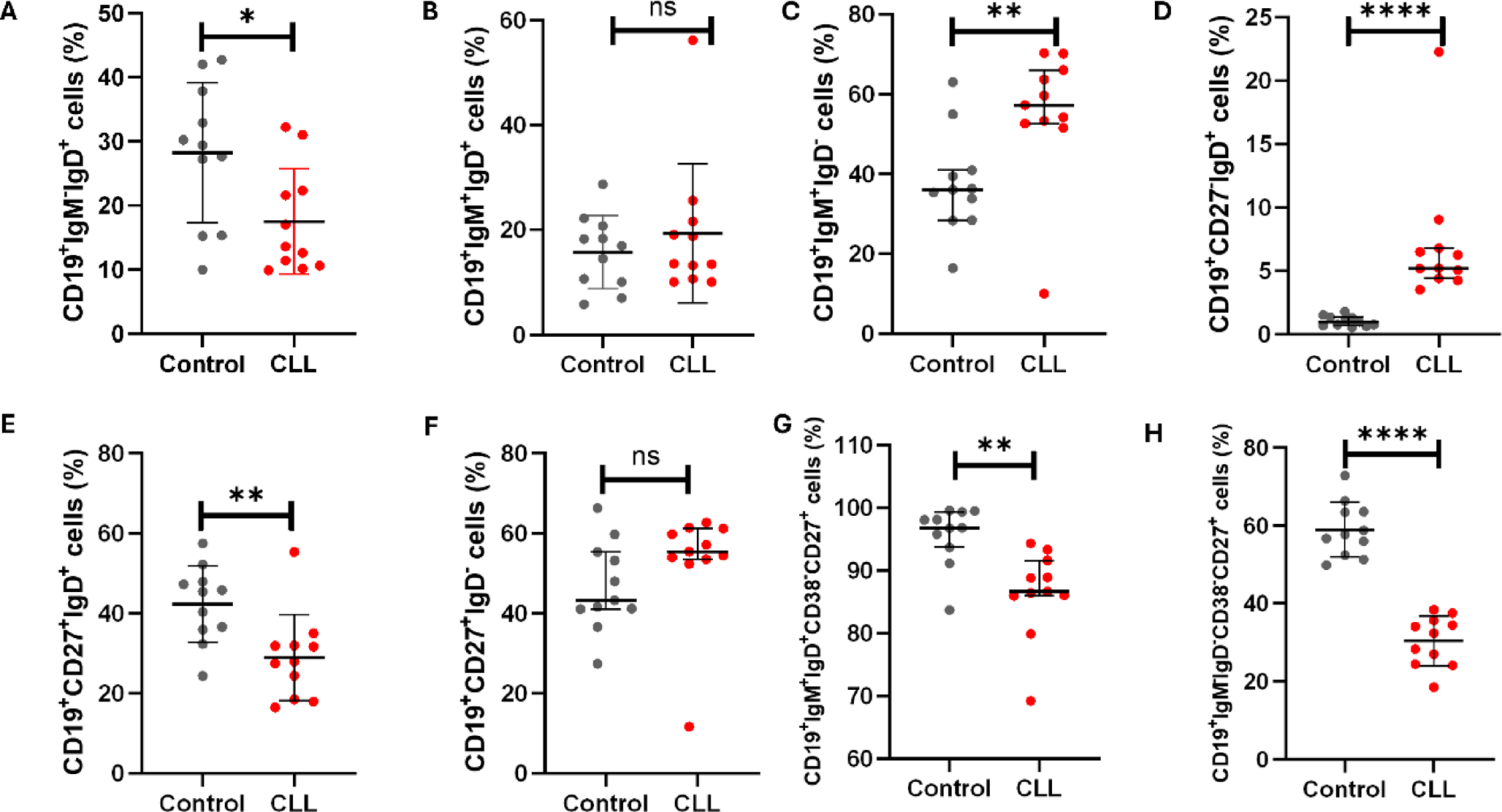
Basal B Cell Subsets levels in patients with CLL. (**A**) illustrates the expression levels of CD19^+^IgM^−^IgD^+^ B cells, (**B**) CD19^+^IgM^+^IgD^+^ B cells, (**C**) CD19^+^IgM^+^IgD^−^ B cells, (**D**) naïve B cells, (**E**) marginal zone B cells, (**F**) CD19^+^CD27^+^IgD^−^ memory B cells, (**G**) non-class switched memory B cells, and (**H**) class switched memory B cells. (**A**,** B**,** E**,** H**) data is presented as the mean ± standard deviation (SD). (**C**,** D**,** F**,** G**) data is presented as the median ± interquartile range (IQR). * *p* = 0.0170, ** *p* < 0.01, and **** *p* < 0.0001 shows the level of significance between groups, ns: not significant

Patients with CLL had significantly decreased levels of CD19^+^IgM^−^IgD^+^ B cells (17.49 ± 8.22) when compared to the control group (28.22 ± 28.22), *p* = 0.0170 (Fig. 2A). The levels of CD19^+^IgM^+^IgD^+^ B cells were comparable between patients with CLL (19.31 ± 13.24) and control group (15.77 ± 6.965), *p* = 0.4420 (Fig. 2B). Notably, the levels of CD19^+^IgM^+^IgD^−^ B cells were significantly increased in patients with CLL, 57.21% (66.02–52.63) when compared to the control group 36.02% (40.97–28.41), *p* = 0.0083 (Fig. 2C). Interestingly, patients with CLL had significantly increased levels of CD19^+^CD27^−^IgD^+^ naïve B cells 5.190% (6.78–4.40) when compared to the control group 0.97% (1.35–0.69), *p* < 0.0001 (Fig. 2D).

As expected, patients with CLL had significantly decreased levels of CD19^+^CD27^+^IgD^+^ marginal zone B cells (28.92 ± 10.81) when compared to the control group (42.30 ± 9.52), *p* = 0.0059 (Fig. 2E). There was no significant difference in CD19^+^CD27^+^IgD^−^ B cells between patients with CLL 55.37% (61.18–53.51) and control group 43.26% (55.29–41.02), *p* = 0.0652 (Fig. 2F). Moreover, patients with CLL had significantly decreased levels of CD19^+^IgM^+^IgD^+^CD38^low^CD27^+^ non-class switched memory B cells 86.63 (91.61 ± 85.96) when compared to the control group 96.83 (99.30-93.69), *p* = 0.0010 (Fig. 2G). Moreover, patients with CLL also had significantly reduced levels of CD19^+^IgM^−^IgD^−^CD38^low^CD27^+^ class switched memory B cells (30.43 ± 6.39) when compared to the control group (58.99 ± 6.99), *p* < 0.0001 (Fig. 2H).

### Association of prognostic markers with the expression levels of B cell subsets

In our multivariable regression model, adjusted for age and sex, there was no association between B2M levels and naïve B cells, marginal zone B cells, class switched memory B cells and non-class switched memory B cells in patients with CLL (Table 3). Moreover, there was no association between CLL-IPI and naïve B cells, marginal zone B cells, class switched memory B cells and non-class switched memory B cells in patients with CLL (Table 3).The Rai stage showed no statistically significant association with naïve B cell levels and marginal zone B cells and significant direct association with class switched memory B cells (Table 3). There was no association between Rai stage and non-class switched memory B cells (Table 3).

**Table 3:** Age, sex adjusted multivariable regression analysis of B cell subsets with prognostic markers in patients with CLL

	B2M			CLL-IPI			Rai stage		
	β (coef)	SE	*p*-value	β (coef)	SE	*p*-value	β (coef)	SE	*p*-value
Naïve B cells	-21.7834	91.455	0.8234	0.0139	0.1140	0.9087	0.4303	0.1752	0.07000
Marginal zone B cells	0.4551	38.253	0.9911	-0.0144	0.0477	0.7779	-0.1808	0.0733	0.0691
Class switched memory B cells	3.98	26.6936	0.8887	-0.0191	0.0333	0.5965	0.1455	0.0511	0.0466 (*)
Non-class switched memory B cells	-10.3283	26.0531	0.712	-0.0049	0.0325	0.8882	-0.0380	0.0499	0.4887
Age	-0.8709	13.2234	0.9507	0.0625	0.0165	0.0192 (*)	-0.0513	0.0253	0.1130
Sex	-154.3047	216.5994	0.5156	0.0266	0.2699	0.9261	-0.8218	0.4149	0.1187

**Fig. 3:**
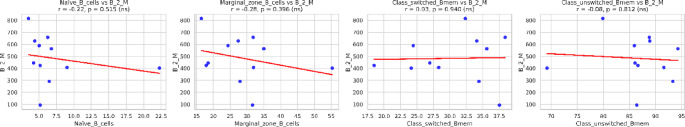
Correlation between B2M and B cell subsets. ns: not significant

The correlation analysis showed no relationship between B2M levels and naïve B cells (*r* = -0.22, *p* = 0.515), marginal zone B cells (*r* = -0.28, *p* = 0.396), class switched memory B cells (*r* = 0.03, *p* = 0.940), class unswitched memory B cells (*r*= -0.08, *p* = 0.812) in patients with CLL (Fig. 3).

**Fig. 4:**
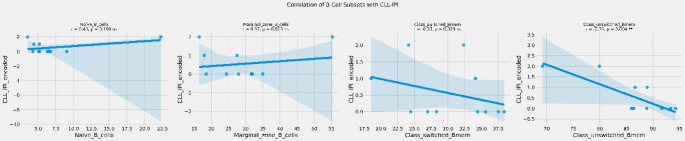
Correlation between CLL-IPI and B cell subsets. ** *p* = 0.004 shows the level of significance between groups, ns: not significant

There was no correlation between CLL-IPI and naïve B cells (*r* = 0.43, *p* = 0.190), marginal zone B cells (*r* = 0.17, *p* = 0.613), class switched memory B cells (*r* = -0.33, *p* = 0.329) in patients with CLL (Fig. 4). There was a significant inverse correlation between CLL-IPI and non-class switched memory B cells (*r* = -0.79, *p* = 0.004) in patients with CLL (Fig. 4).

**Fig. 5:**
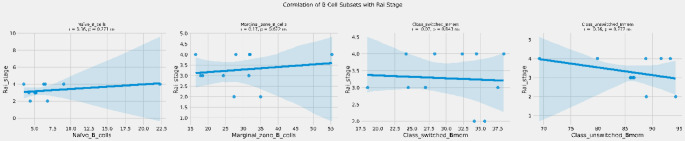
Correlation between Rai stage and B cell subsets. ns: not significant

 There was no correlation between Rai stage and naïve B cells (*r* = 0.36, *p* = 0.271), marginal zone B cells (*r* = 0.17, *p* = 0.622), class switched memory B cells (*r* = -0.07, *p* = 0.843), non-class switched memory B cells (*r*= -0.36, *p* = 0.277) in patients with CLL (Fig. 5).

## Discussion

The aim of this study was to investigate the prognostic value of B cell subsets profiles in patients with CLL. In our study, we observed increased levels of naïve B cells. In contrary, previous studies demonstrated decreased levels of naïve B cells in patients with CLL [19, 20]. Furthermore, reduction of naïve B cells has been shown to be associated with disease severity [20]. The increase we observed could reflect disease-associated immune remodelling or impaired B cell maturation patterns in progressive CLL. The CLL microenvironment disrupts the development of mature functionally competent B cells [21]. This may lead to the failure of naive B cells to mature properly. Therefore, since our study measured total naïve B cells without distinguishing between normal and malignant populations, the observed increase may reflect an accumulation of normal healthy naïve B cells rather than malignant cells. However, given the small sample size in our study, further investigation in a larger cohort is warranted to confirm this trend and clarify its biological and clinical implications.

As expected, our study demonstrated reduced levels of marginal zone B cells, non-class switched memory B cells and class switched memory B cells. Our findings are consistent with previous studies showing reduced levels of CD27^+^ memory B cells in patients with CLL [14, 20, 22]. However, it is important to note that those studies primarily characterized malignant CD5⁺ memory B cells. Furthermore, our study demonstrated the significant value of class switched memory B cells by demonstrating a direct association with Rai stage. CLL derives from a continuum of maturation states, with methylation patterns reflecting stages similar to normal B cell development [23]. Since class-switched memory B cells are a mature subset, their reduction could potentially associate with disease progression.

Interestingly, our study showed no significant correlation between marginal zone B‑cell levels and Rai stage. Notably, we detected an inverse correlation between non-class switched memory B cells and the CLL‑IPI score, suggesting that reduced levels of these cells may be associated with poor prognosis. These findings align with previous immunophenotypic study showing that circulating memory B cells consist of two distinct populations, IgM^hi^ cells resembling germinal center-derived marginal zone B cells, and IgM^lo^ cells representing less differentiated memory subsets [24]. Therefore, our data suggests non-class switched memory B cells with IgM^hi^ subpopulation, closely relate to marginal zone B cells. These cells may be preferentially reduced in patients with more aggressive disease profiles.

A limitation of our study is that the analysis was performed on total B cells, encompassing both normal and malignant populations. In addition, our small sample size limits the generalization of the reported study findings. Future studies need to include larger, well-characterised cohorts and employ single-cell analytical approaches to distinguish malignant from normal B cells. These approaches are necessary to achieve a more precise understanding of the biological and prognostic relevance of specific B cell subsets in CLL. Furthermore, because this study was cross-sectional and included treatment-naïve patients at a single time point, longitudinal data on overall survival (OS) and time to treatment (TTT) were not available. Consequently, it was not possible to directly assess the association between memory B cell subsets and clinical outcomes. Future longitudinal studies are necessary to determine the prognostic significance of these subsets in predicting disease progression and treatment response.

## Conclusion

Memory B cell subpopulations are markedly reduced in patients with CLL, and their altered distribution may hold prognostic significance in disease progression and risk stratification.

## Supplementary Information

Below is the link to the electronic supplementary material.

Supplementary Material 1

## Data Availability

All data supporting the findings of this study are included in the article and its Supplementary Materials. Additional data generated during the study are available from the corresponding author upon request.
