# Supplementary material for: Prognostic value of memory B cell subpopulation in patients with chronic lymphocytic leukemia
Source: Clin Exp Med. 2025 Dec 24;26(1):80. doi: 10.1007/s10238-025-02002-5 (PMC12769603; doi:10.1007/s10238-025-02002-5)
Supplement: Supplementary file 1 — Supplementary Material 1 [file 10238_2025_2002_MOESM1_ESM.pdf]

Supplementary Materials

Table S1: Biological characteristics of patients with CLL

Patient ID	Age	Sex	Rai stage	FISH status	β2 microglobulin	IGHV status
CLL-1	57	F	IV	del(11q22)	0.9	Mutated
CLL-2	65	M	III	del(13q14)	0.1	Mutated
CLL-3	68	M	II	del(13q14)	0.8	Mutated
CLL-4	83	F	IV	del(17p13)	1.0	Mutated
CLL-5	55	M	III	del(13q14)	0.6	Mutated
CLL-6	48	M	III	del(13q14)	0.8	Mutated
CLL-7	67	F	III	del(13q14)	0.6	Mutated
CLL-8	57	F	IV	del(11q22)	0.5	Mutated
CLL-9	57	M	II	del(13q14)	0.8	Mutated
CLL-10	55	M	IV	del(11q22)	0.4	Mutated
CLL-11	86	F	IV	del(17p13)	0.5	Mutated

Fluorescence compensation was performed using single-stained DURAClone compensation controls (Beckman Coulter). Compensation matrices were adjusted to ensure minimal spectral overlap across all six fluorochromes before data acquisition.

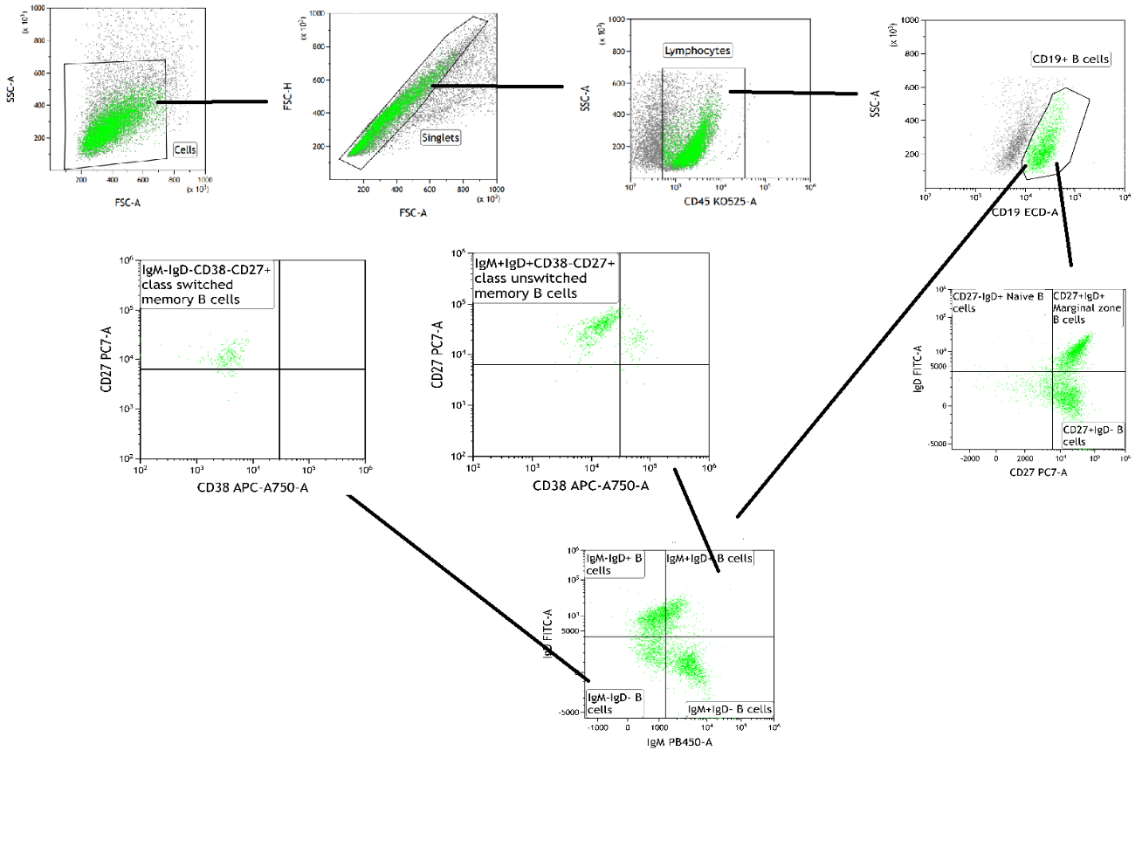

Figure S1: Representative gating strategy for identification of B-cell subsets in PBMCs from patient with CLL.
